# Averaging real-time impedance enhances the prediction of steam pop risk and lesion characteristics

**DOI:** 10.1093/europace/euaf315

**Published:** 2025-12-09

**Authors:** Hidehiro Iwakawa, Masateru Takigawa, Junji Yamaguchi, Ryosuke Kato, Masaki Honda, Ryo Tateishi, Miho Negishi, Iwanari Kawamura, Kentaro Goto, Kensuke Ihara, Takuro Nishimura, Kazuya Yamao, Susumu Tao, Sayaka Suzuki, Takehiro Iwanaga, Iichiro Onishi, Shinsuke Miyazaki, Hiroyuki Watanabe, Tetsuo Sasano

**Affiliations:** Department of Cardiovascular Medicine, Institute of Science Tokyo, 1-5-45 Yushima, Bunkyo-ku, Tokyo 113-8519, Japan; Department of Cardiovascular Medicine, Akita University Graduate School of Medicine, Akita, Japan; Department of Cardiovascular Medicine, Institute of Science Tokyo, 1-5-45 Yushima, Bunkyo-ku, Tokyo 113-8519, Japan; Division of Advanced Arrhythmia Research, Institute of Science Tokyo, 1-5-45 Yushima, Bunkyo-ku, Tokyo 113-8519, Japan; Department of Cardiovascular Medicine, Institute of Science Tokyo, 1-5-45 Yushima, Bunkyo-ku, Tokyo 113-8519, Japan; Department of Clinical and Diagnostic Laboratory Science, Institute of Science Tokyo, Tokyo, Japan; Department of Cardiovascular Medicine, Institute of Science Tokyo, 1-5-45 Yushima, Bunkyo-ku, Tokyo 113-8519, Japan; Department of Cardiovascular Medicine, Akita University Graduate School of Medicine, Akita, Japan; Department of Cardiovascular Medicine, Institute of Science Tokyo, 1-5-45 Yushima, Bunkyo-ku, Tokyo 113-8519, Japan; Department of Cardiovascular Medicine, Institute of Science Tokyo, 1-5-45 Yushima, Bunkyo-ku, Tokyo 113-8519, Japan; Department of Cardiovascular Medicine, Institute of Science Tokyo, 1-5-45 Yushima, Bunkyo-ku, Tokyo 113-8519, Japan; Department of Cardiovascular Medicine, Institute of Science Tokyo, 1-5-45 Yushima, Bunkyo-ku, Tokyo 113-8519, Japan; Department of Cardiovascular Medicine, Institute of Science Tokyo, 1-5-45 Yushima, Bunkyo-ku, Tokyo 113-8519, Japan; Division of Advanced Arrhythmia Research, Institute of Science Tokyo, 1-5-45 Yushima, Bunkyo-ku, Tokyo 113-8519, Japan; Department of Cardiovascular Medicine, Institute of Science Tokyo, 1-5-45 Yushima, Bunkyo-ku, Tokyo 113-8519, Japan; Department of Cardiovascular Medicine, Institute of Science Tokyo, 1-5-45 Yushima, Bunkyo-ku, Tokyo 113-8519, Japan; Department of Cardiovascular Medicine, Institute of Science Tokyo, 1-5-45 Yushima, Bunkyo-ku, Tokyo 113-8519, Japan; Department of Cardiovascular Medicine, Institute of Science Tokyo, 1-5-45 Yushima, Bunkyo-ku, Tokyo 113-8519, Japan; Japan Small Animal Medical Center, Saitama, Japan; Animal Research Facilities, Bioscience Center, Research Infrastructure Management Center, Institute of Science Tokyo, Tokyo, Japan; Department of Pathology, Institute of Science Tokyo, Tokyo, Japan; Department of Cardiovascular Medicine, Institute of Science Tokyo, 1-5-45 Yushima, Bunkyo-ku, Tokyo 113-8519, Japan; Division of Advanced Arrhythmia Research, Institute of Science Tokyo, 1-5-45 Yushima, Bunkyo-ku, Tokyo 113-8519, Japan; Department of Cardiovascular Medicine, Akita University Graduate School of Medicine, Akita, Japan; Department of Cardiovascular Medicine, Institute of Science Tokyo, 1-5-45 Yushima, Bunkyo-ku, Tokyo 113-8519, Japan

**Keywords:** Catheter ablation, Impedance, In vivo, Lesion size, Steam pop

## Abstract

**Aims:**

A novel impedance filtering function that averages impedance values was developed to mitigate cardiac and respiratory oscillations. We aimed to evaluate the clinical significance of averaging real-time impedance in predicting steam pops (SPs) and lesion characteristics.

**Methods and results:**

Radiofrequency (RF) ablation was performed in 20 swine using a flexible-tip temperature-controlled power regulation catheter. Both unfiltered and filtered (averaged) impedance values were recorded using the EnSite™ X system. For each RF application, absolute (ΔImp-drop) and relative (%Imp-drop) impedance drops were quantified. Associations between impedance parameters and SP occurrence, atrial lesion transmurality, and ventricular lesion dimensions were evaluated. Among 959 lesions, SPs occurred in 36 applications (3.8%), all within the ventricles. Notably, 6 SPs occurred within 90 s despite RF power ≤ 40 W, with 4 during left ventricular ablation under low systolic blood pressure (<40 mmHg). Lesions with SPs exhibited significantly greater unfiltered and averaged ΔImp-drop and %Imp-drop (all *P* < 0.001). Averaged %Imp-drop showed the highest predictive value for SPs (AUC = 0.93), with a 20.9% cut-off yielding 88.9% sensitivity and 85.5% specificity. The time to reach the initial 10%, 15%, and 20% reduction in averaged %Imp-drop was not associated with SP occurrence. Both unfiltered and averaged impedance drops correlated with atrial transmural lesion formation. Averaged impedance drops significantly improved estimation of lesion depth, surface area, and volume compared with unfiltered values (*P* < 0.01).

**Conclusion:**

The averaged relative impedance drop demonstrated the strongest association with SP occurrence, and averaging impedance provided a more accurate assessment of lesion characteristics than unfiltered measurements.

## Graphical Abstract

**Graphical Abstract:**
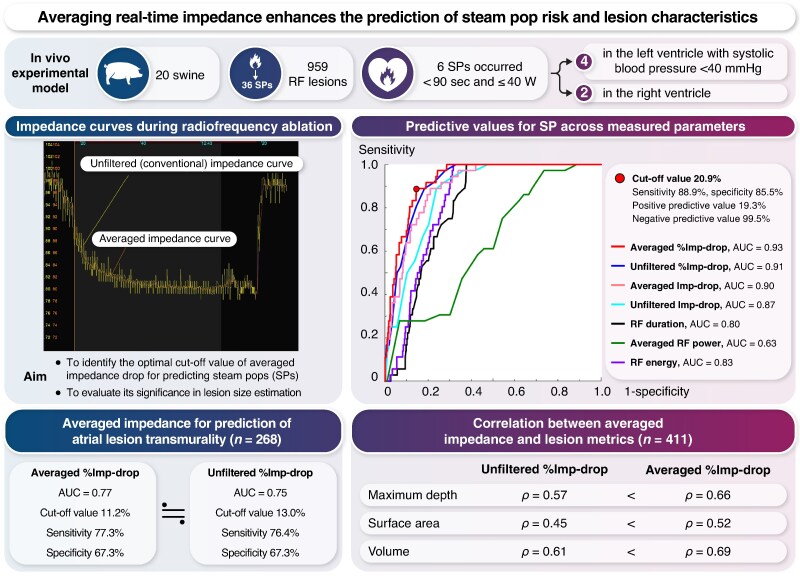

What’s new?

- Averaged relative impedance drop best predicts steam pops (SPs) compared with other parameters, including unfiltered impedance drop, RF power, duration, and energy.
- Early rate of impedance decline may not be associated with SP occurrence.
- Averaged impedance correlates well with lesion transmurality and size, outperforming unfiltered values.
- Low systemic perfusion was associated with increased SP risk, even at moderate RF power.

## Introduction

Pulsed-field ablation (PFA) has recently emerged as a promising technology to improve the efficacy and safety of atrial fibrillation (AF) ablation, especially in pulmonary vein isolation.^1^ Nevertheless, radiofrequency (RF) ablation continues to play an essential role in specific scenarios, supported by substantial evidence, such as complex atrial tachycardias involving epicardial structures and ventricular arrhythmias requiring deeper lesions.^2,3^ In addition, the high start-up cost of PFA may limit its widespread adoption across healthcare systems,^4^ particularly in resource-limited settings. Thus, the established role of RF ablation, supported by its long-standing safety profile,^5^ is unlikely to diminish in the foreseeable future.

Despite advances in catheter design, energy control, and lesion-quality metrics,^6–8^ RF ablation remains inherently associated with intramyocardial heating, and steam pops (SPs) still occur. SPs can result in myocardial rupture, leading to serious complications such as cardiac perforation and tamponade.^9^ Macroscopically, lesions with SPs are characterized by tissue perforation and crater formation, while histopathological assessment reveals extensive coagulation necrosis and cellular dissolution (see Supplementary material online, *Figure S1*). Therefore, enhancing the accuracy of SP prediction is clinically relevant.

Impedance drop during RF application correlates with lesion size, and an excessive drop has been considered a predictor of SPs.^10–12^ However, impedance values constantly fluctuate with cardiac and respiratory motion, and these physiological artifacts can hinder accurate monitoring. To address this, new algorithms have recently been developed to average fluctuations in the impedance signal, potentially providing a more reliable estimate of tissue response. Yet, the clinical utility of averaged impedance for predicting SPs and lesion characteristics has not been systematically evaluated.

This study aimed to identify the optimal cut-off value of averaged impedance drop for predicting SPs and to evaluate whether it improves the accuracy of lesion size estimation compared with conventional metrics, using a porcine *in vivo* model.

## Methods

### In vivo experimental model using swine and ablation protocols

The protocol for the *in vivo* experiment was approved by the Institutional Animal Care and Use Committees of Institute of Science Tokyo (A2024-125C). Twenty castrated swine (aged 2.9–4.2 months; weight 48.5–59.0 kg) were sedated with an intramuscular injection of ketamine hydrochloride (10 mg/kg) and xylazine (2 mg/kg). After sedation, each swine was intubated, following the inhalation of isoflurane, and anesthesia was maintained with 2–3% isoflurane. Ventilation was provided using a respirator (Carestation/Carescape, GE Healthcare, Chicago, IL), with room air supplemented with oxygen. An intravenous sheath was inserted via the internal jugular vein for drug and fluid infusion. Intravenous amiodarone was administered to prevent ventricular arrhythmias. Arterial pressure and blood gases were periodically monitored, with ventilator parameters adjusted to maintain blood gas levels within physiological ranges.

A decapolar catheter was positioned in the coronary sinus (CS) through the internal jugular vein. After the transseptal puncture, an Agilis™ deflectable sheath (Abbott, Abbott Park, IL) was advanced to the left atrium (LA) to approach the left ventricle (LV) under the fluoroscopic guidance. With support of the deflectable sheath, four-chamber mapping was conducted using the Advisor™ HD Grid catheter and EnSite™ X EP System (Abbott, Abbott Park, IL) during CS pacing. RF ablations were then performed using the contact-force (CF) sensing ablation catheter (TactiFlex™ SE, Abbott, Abbott Park, IL), with a target CF of 10 to 15 g at varying RF powers (30–50 W) and durations. Briefly, in the atria, RF was applied for at least 5 s and up to 50 s; in the right ventricle (RV), for at least 5 s and up to 80 s; and in the LV, for at least 5 s and up to 300 s. Importantly, RF protocol included a subset of applications with durations longer than those typically used in routine clinical practice. These extended applications were deliberately employed to assess the association between study parameters and SP occurrence across a broad range and non-standard clinical scenarios. This design facilitated the accrual of a sufficient number of SP events under varied conditions. We do not advocate such prolonged or non-standard ablations in patient care; they were undertaken solely to stress-test the potential performance limits of the catheter system.

### Ablation catheter

The TactiFlex™ SE catheter features a 4-mm 8Fr tip with laser-cut kerfs on the side. A calibrated roller pump (Cool Point™, Abbott, Abbott Park, IL) connected to the catheter delivered normal saline at a rate of 13 mL/min during RF applications. A single thermocouple embedded within the tip for temperature monitoring, which is situated 0.3 mm from the tip. A power-controlled ablation mode with a temperature cut-off of 43 °C was used.

### Unfiltered and averaged impedance measurements

**Figure 1:**
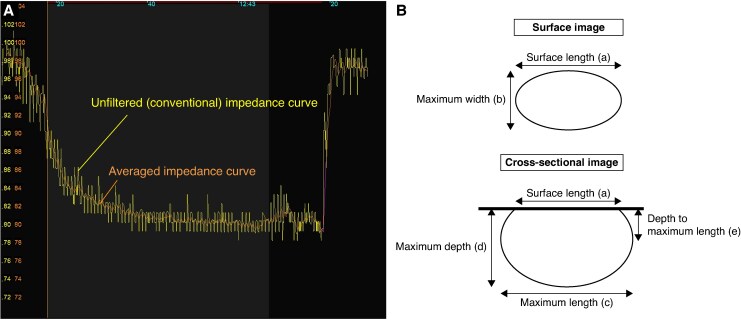
(*A*) Representative unfiltered (*yellow*) and averaged (*orange*) impedance curves during RF ablation. The *X*-axis and *Y*-axis represent time and impedance values, respectively. The gray and black backgrounds indicate the duration of RF application being on and off. The unfiltered impedance values frequently fluctuate by 2–4 Ω, while the averaged impedance values show a steady decrease during RF application. (*B*) Scheme of lesion measurements.

Both unfiltered (or conventional) and averaged impedance values, along with time-dependent changes in RF power, duration, CF, and tip temperature during each RF application were recorded. The absolute impedance drop (ΔImp-drop), calculated by subtracting the minimum impedance from the initial impedance value and the relative impedance drop (%Imp-drop), determined by dividing ΔImp-drop by the initial impedance value, were automatically measured using the EnSite™ X EP System. The real-time averaged impedance was calculated by applying a one-second moving average filter to the raw impedance signal recorded by Ampere RF generator (Abbott, Abbott Park, IL), thereby attenuating cardiac motion-related oscillations (∼1–2 Hz) and yielding a smoothed impedance profile. SP was defined as audible pop during RF application. We investigated the association between the occurrence of SPs and the duration required for the initial 10–20% impedance drop. *Figure 1A* shows the representative time-dependent unfiltered and averaged impedance changes during the RF applications.

### Lesion size measurements

Regarding *in vivo* experimental model, swine were sacrificed and endomyocardial lesions were measured following RF deliveries. The lesion border was defined as the location of a change in tissue color. The lesions in which SPs (defined as audible pops) occurred were excluded from this study. The myocardium was cross-sectioned along the surface length at the level of each lesion. Each lesion was measured with a digital caliper with a resolution of 0.1 mm by one observer who was blinded to the lesion protocol.

The lesion transmurality in the atrial walls was assessed in tissue with a thickness of ≤ 3 mm, based on prior experimental studies.^13,14^ On gross pathological assessment, lesions with an epicardial lesion length of ≥ 4 mm and width ≥ 3 mm, corresponding to a lesion area ≥ 9.42 cm^2^, were defined as transmural.

The surface length (a), surface width (b), maximum length (c), maximum depth (d), and depth to the maximum length (e) of the created lesion were measured as shown in *Figure 1B*. Lesion surface area and lesion volume were calculated from the following formulae: Lesion surface area = π × a/2 × b/2, Lesion volume = (1/6) × π × (c^2^×d + e×a^2^/2).^15–17^

### Statistical analysis

Continuous variables are expressed as mean ± standard deviation or median and 25th percentile–75th percentile. For categorical variables, data are described as numbers and percentages. To compare two groups, parametric data were analyzed using Student’ *t*-tests and non-parametric data using Mann–Whitney *U* tests. For the subgroup analysis, a Bonferroni correction was applied to control the α level (statistical significance set at *P* < 0.025). Receiver operating characteristic (ROC) curve analysis was performed to assess the optimal cut-off value for predicting SP and transmural atrial lesions. The DeLong test was performed to compare the area under the curve (AUC) among the measured parameters. Spearman’s rank correlation analysis was used to assess the correlation between variables. Fisher’s *Z* transformation was performed to compare the strength of correlation.^18^ Statistical significance was defined as two-sided *P*-values < 0.05. Statistical analyses were performed using StatFlex software version 7.1 (Artech, Osaka, Japan).

## Results

### Predictive values for SPs

**Figure 2:**
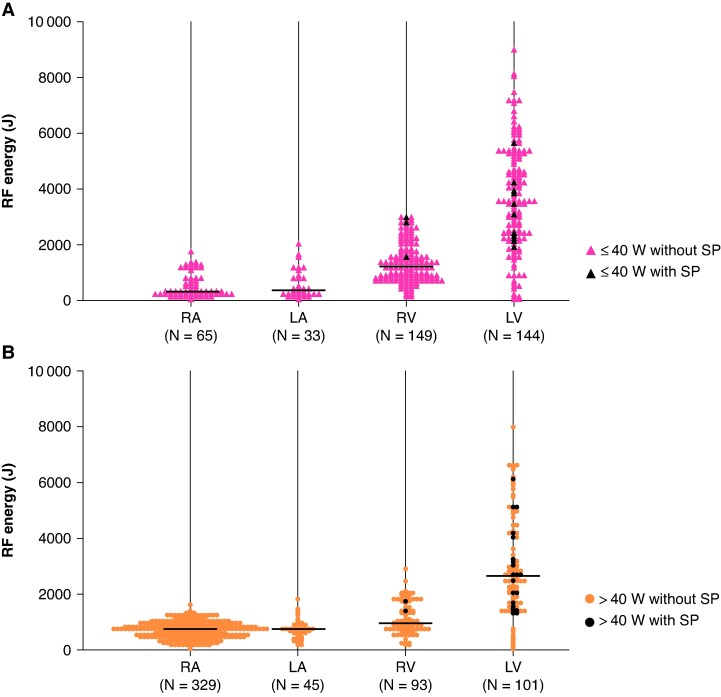
Association between lesion anatomical location and delivered RF energy. Swarm plots show the RF energy delivered to lesions in the RA, LA, RV, and LV, stratified by power setting: ≤ 40 W (*A*) and > 40 W (*B*). Pink triangles, black triangles, orange circles, and black circles represent lesions at ≤ 40 W without SPs, at ≤ 40 W with SPs, at > 40 W without SPs, and at > 40 W with SPs, respectively.

*Figure 2* shows associations between anatomical locations of the lesions and corresponding RF energies. SPs were observed in 36 out of 959 lesions (3.8%). No SPs occurred in the atrium (*n* = 472), whereas all SPs were observed during ventricular ablations (36 out of 487 lesions, 7.4%). Six SPs occurred within 90 s during ablation at RF power settings of ≤ 40 W. These were performed either in the LV when the swine’s systolic blood pressure was below 40 mmHg (*n* = 4) or in the RV (*n* = 2).

*Table 1* summarizes the ablation parameters with and without SPs. The unfiltered ΔImp-drop, averaged ΔImp-drop, unfiltered %imp-drop, and averaged %Imp-drop were significantly higher in lesions with SPs (*P* < 0.001 for all). Averaged RF power (without vs. with SPs, 41.0 [34.0–47.0] W vs. 44.0 [39.5–49.0] W, *P* = 0.007) and RF durations (without vs. with SPs, 21.0 [15.0–42.0] sec vs. 62.5 [38.5–88.5] sec, *P* < 0.001) were significantly larger with SPs. By contrast, there were no significant differences in the CF, initial impedance values, and averaged tip temperatures with and without SPs. These trends were consistent when assessed only in the ventricular lesions (see Supplementary material online, *Table S1*).

**Table 1:** Ablation parameters with and without SPs

	SP (−)	SP (+)	*P*-values
	*n* = 923	*n* = 36	
Predefined RF power, *n* (%)	30 W: 116 (12.6%)	30 W: 1 (2.8%)	0.002*
	35 W: 116 (12.6%)	35 W: 3 (8.3%)	
	40 W: 151 (16.4%)	40 W: 10 (27.8%)	
	45 W: 113 (12.2%)	45 W: 11 (30.6%)	
	50 W: 427 (46.3%)	50 W: 11 (30.6%)	
Averaged RF power, W	41.0 [34.0–47.0]	44.0 [39.5–49.0]	0.007*
Averaged contact force, g	13.0 [10.0–16.0]	12.5 [10.0–16.0]	0.37
Initial impedance, Ω	106 [100–114]	105 [103–110]	0.58
Averaged initial impedance, Ω	105 [99–113]	104 [100–110]	0.66
ΔImp-drop, Ω	18.0 [14.0–23.0]	27.5 [24.0–30.5]	<0.001*
Averaged ΔImp-drop, Ω	15.3 [11.6–19.9]	25.1 [22.6–28.1]	<0.001*
%Imp-drop, %	17.0 [13.0–21.0]	26.5 [24.0–28.0]	<0.001*
Averaged %Imp-drop, %	14.4 [11.3–18.3]	24.1 [22.2–26.6]	<0.001*
Duration, sec	21.0 [15.0–42.0]	62.5 [38.5–88.5]	<0.001*
Energy, J	905 [558–1752]	2581 [1726–3658]	<0.001*
Averaged temperature, °C	36.0 [34.0–39.0]	36.0 [34.0–38.0]	0.79
Reach maximum temperature, *n* (%)	236 (25.6%)	8 (22.2%)	0.65

Abbreviations: Imp, impedance; RF, radiofrequency; SP, steam pop. *P < 0.05.

**Figure 3:**
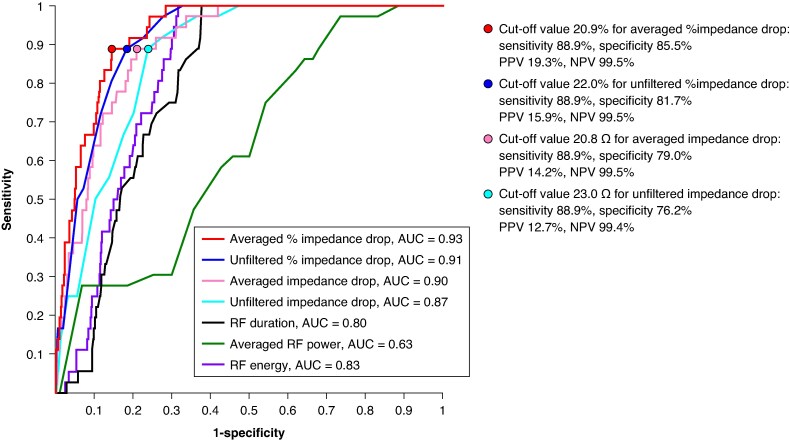
ROC curves for predicting SP. NPV, negative predictive value; PPV, positive predictive value.

*Figure 3* depicts ROC curves for predicting SPs. The AUC values of unfiltered ΔImp-drop, averaged ΔImp-drop, unfiltered %imp-drop, averaged %Imp-drop, averaged RF power, duration, and energy were 0.87, 0.90, 0.91, 0.93, 0.63, 0.80, and 0.83, respectively. The averaged ΔImp-drop significantly improved the predictive value for SPs compared with the unfiltered measure (AUC = 0.91 vs. 0.87, *P* = 0.03). The averaged %Imp-drop demonstrated the highest AUC value among all measured parameters and showed a trend toward a higher in the AUC value compared with the unfiltered %Imp-drop, although the difference was not statistically significant (AUC = 0.93 vs. 0.91, *P* = 0.13). When the cut-off value was set at 20.9% for averaged %Imp-drop, the sensitivity, specificity, positive predictive value (PPV), and negative predictive value (NPV) for SPs was 88.9%, 85.5%, 19.3%, and 99.5%, respectively.

To assess the association between the rate of the initial impedance drop and SPs, we conducted a subgroup analysis limited to lesions with an averaged %Imp-drop ≥ 20.9% (*n* = 166). *Table 2* summarizes ablation parameters for lesions with and without SPs in this subset. The time required to achieve the initial 10%, 15%, and 20% reductions in averaged %Imp-drop did not differ between the two groups. Although the difference did not reach significance, the median averaged RF power tended to be higher in lesions with SPs than in those without (44.5 [40.0–49.0] W vs. 40.5 [35.0–46.0] W; *P* = 0.04).

**Table 2:** Subgroup analysis of ablation parameters with and without SPs among lesions with an averaged %imp-drop at or above the cut-off value

	SP (−)	SP (+)	*P*-values
	*n* = 134	*n* = 32	
Predefined RF power, *n* (%)	30 W: 12 (9.0%)	30 W: 1 (3.1%)	0.53
	35 W: 19 (14.2%)	35 W: 3 (9.4%)	
	40 W: 33 (24.6%)	40 W: 7 (21.9%)	
	45 W: 30 (22.4%)	45 W: 11 (34.4%)	
	50 W: 40 (29.9%)	50 W: 11 (31.3%)	
Averaged RF power, W	40.5 [35.0–46.0]	44.5 [40.0–49.0]	0.04
Averaged contact force, g	13.0 [10.0–16.0]	12.5 [10.0–16.0]	0.31
Duration required for initial 10% of averaged %Imp-drop, sec	3.0 [2.0–6.0]	3.0 [2.0–4.3]	0.33
Duration required for initial 15% of averaged %Imp-drop, sec	14.0 [7.0–24.0]	12.0 [9.0–18.0]	0.33
Duration required for initial 20% of averaged %Imp-drop, sec	33.0 [17.0–53.0]	30.0 [18.8–43.0]	0.28
Duration, sec	64.5 [39.0–121.0]	62.5 [38.5–92.5]	0.42
Energy, J	2811 [1589–4591]	2581 [1726–3937]	0.66
Averaged temperature, °C	36.0 [34.0–38.0]	36.0 [34.0–37.0]	0.41
Reach maximum temperature, *n* (%)	34 (25.4%)	5 (15.6%)	0.24

Bonferroni corrected *P* < 0.025.

### Predictors for transmural atrial lesions

**Figure 4:**
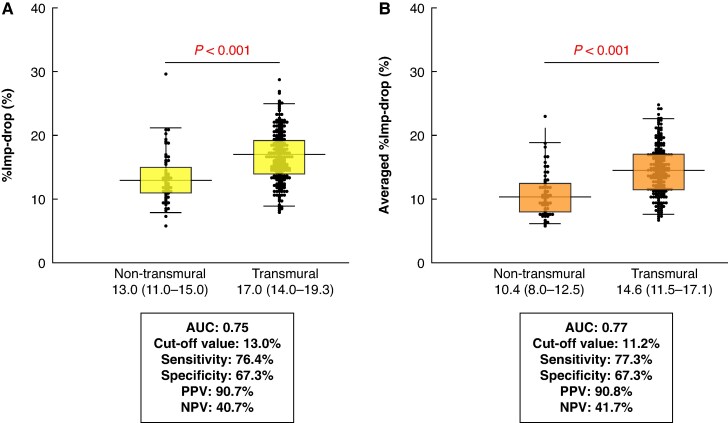
Unfiltered and averaged impedance drops in non-transmural and transmural lesions. Box plots illustrate the distribution for (*A*) unfiltered %Imp-drop and (*B*) averaged %Imp-drop. The area under the receiver operating characteristic curve (AUC), optimal cut-off values, sensitivity, specificity, PPV, and NPV are shown below each plot.

Lesion transmurality was assessed in 268 isolated atrial lesions [right atrium (RA), *n* = 198; LA, *n* = 70]. Transmurality was observed in 209 of 268 lesions (78.0%). The unfiltered and averaged %Imp-drop was significantly larger in transmural lesions than in non-transmural lesions (*P* < 0.001) (*Figure 4*). Although the absolute AUC was slightly higher for averaged %Imp-drop than for unfiltered one (averaged %Imp-drop, AUC = 0.77; unfiltered %Imp-drop, AUC = 0.75), the differences did not reach significance (*P* = 0.58). These observations were consistent for unfiltered and averaged ΔImp-drop (see Supplementary material online, *Figure S2*).

### Averaged impedance for lesion size estimation

**Figure 5:**
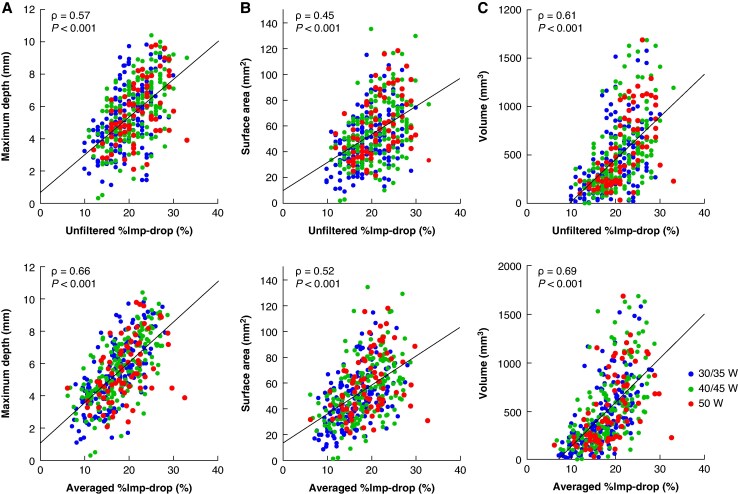
Correlations of unfiltered (*upper*) and averaged (*lower*) %imp-drop with lesion metrics. Scatter plots show relationships with maximum depth (*A*), surface area (*B*), and volume (*C*). Blue, green, and red dots represent lesions created with RF power of 30–35 W, 40–45 W, and 50 W, respectively.

Correlations of both unfiltered and averaged impedance drops with lesion size were assessed, excluding transmural or insufficient ventricular lesions, as well as lesions with SPs (*n* = 411). Both unfiltered and averaged %Imp-drop significantly correlated with maximum depth (unfiltered, *ρ* = 0.57; averaged, *ρ* = 0.66), surface area (unfiltered, *ρ* = 0.45; averaged, *ρ* = 0.52), and volume (unfiltered, *ρ* = 0.61; averaged, *ρ* = 0.69) (*Figure 5*). Averaging significantly increased the strength of correlation with the lesion maximum depth, surface area, and volume compared with conventional %Imp-drop (*P* < 0.01). Similarly, averaged ΔImp-drop showed better correlations with lesion size than unfiltered one, although the strength of correlation was generally more modest than that of averaged %Imp-drop (see Supplementary material online, *Figure S3*).

## Discussion

### Major findings

We explored the significance of averaged impedance measurements for predicting SPs and lesion formation, using a large number of atrial and ventricular RF lesions created with the TactiFlex™ ablation catheter in an *in vivo* model. Our findings were as follows:

1. The averaged %Imp-drop demonstrated the highest predictive value for SP among all measured parameters including ΔImp-drop, power, RF duration, and energy.
2. When the cut-off value was set at 20.9% for averaged %Imp-drop, the sensitivity and specificity for predicting SPs were 88.9% and 85.5%, respectively.
3. The initial rate of decline from baseline in averaged baseline impedance was not associated with SP occurrence.
4. Both averaged and unfiltered impedance drops were significantly correlated with the formation of atrial transmural lesions.
5. Averaged impedance metrics significantly improved the predictive accuracy for lesion size compared with unfiltered impedance measurements.
6. SP risk should be carefully considered under low systemic perfusion, such as during hypotension, even when RF power is reduced.

### Evolution of techniques, current position, and remaining challenges of RF catheter ablation

Over the past two decades, catheter design and energy-delivery strategies for RF ablation of AF have evolved substantially, and clinical indications have broadened.^1,19^ Early non-irrigated, temperature-controlled catheters were limited by tip overheating, preventing effective energy delivery and increasing thrombus risk. The advent of open-irrigated, CF-sensing catheters enabled higher and more consistent RF power delivery with reduced thrombus formation and spurred development of lesion-quality indices.^6–8^ Building on these developments, newer catheters incorporate technologies that enable reliable tissue-surface temperature monitoring, even under irrigation, during high power, short-duration ablation, thereby achieving adequate lesion size while suppressing the incidence of SPs.^20^ These advances in catheter design are now widely used for RF ablation of other arrhythmias.

Despite this progress, a mismatch between electrode-tip and tissue temperatures during irrigation can allow deeper tissue heating, which, under certain conditions, may lead to SPs within the myocardium.^21^ Notably, SPs continue to be observed even with contemporary open-irrigated, temperature-controlled catheters.^22,23^ Although the incidence of SPs is relatively low,^9^ their clinical consequences can be devastating. An analysis of post-market surveillance data identified that 8–16% of patient-related adverse events were due to SPs, resulting in cardiac perforation or death.^22^ This underscores the continuing need for reliable intraprocedural metrics to guide safe energy delivery.

Within this context, our study demonstrated that impedance averaging attenuates physiological fluctuations, providing a more stable signal for predicting SPs and assessing lesion formation. By refining real-time risk assessment, this approach may enhance procedural safety and support clinical decision-making in contemporary RF ablation practice.

### Impact of averaging impedance on prediction of SP and lesion formation

This study demonstrated that averaged impedance metrics had greater predictive value for SPs and lesion formation than conventional impedance measures. The NPV of 99.5% is clinically valuable, as it suggests that when the averaged %Imp-drop remains below the cut-off value of 20.9%, the probability of an SP is very low, potentially allowing operators to continue RF delivery. The modest PPV is primarily attributable the low prevalence of SPs (3.8%) in this study, even under deliberately aggressive ablation protocols.

We previously reported that %Imp-drop was a significant predictor of SP, and a cut-off value of 20% demonstrated sensitivity and specificity of 100% and 89.6%, respectively, using the TactiFlex™ ablation catheter in an ex vivo model.^12^ Although sensitivity and specificity were slightly lower in this *in vivo* model than in the previous ex vivo data, the usefulness of a 20% %Imp-drop threshold for predicting SP was validated, suggesting that setting the cut-off value for averaged %Imp-drop around 20% is plausible in clinical practice. Nonetheless, because the PPV was modest, exceeding this threshold should be viewed as a risk alert rather than a deterministic trigger, since SPs will not invariably occur once the cut-off is crossed.

With respect to efficacy of the cut-offs, prior studies have proposed impedance-drop thresholds of at least 10–15 Ω for achieving successful lesion creation,^1,24^ which are lower than the best cut-off of 20.8 Ω for averaged ΔImp-drop in predicting SP occurrence (*Figure 3*). In addition, based on the correlation between %Imp-drop and lesion depth, the median lesion depth was estimated at 6.3 [5.4–7.2] mm when the target %Imp-drop reached the cut-off of 20.9%. This depth may be sufficient to achieve atrial transmural lesions and to modify the ventricular substrate at a certain depth from the endomyocardium.^13^ Therefore, these cut-offs for both averaged %Imp-drop and ΔImp-drop are unlikely to overly restrict RF energy delivery needed for adequate lesion formation. Accordingly, in clinical settings where deeper lesions are required, it may be reasonable, while carefully avoiding conditions that diminish convective cooling (e.g. hypotension), to eschew very high power and instead consider longer applications at ≤ 40 W; however, this approach requires further study.

We evaluated the predictive value of averaged impedance drops for atrial transmural lesions in this study. While averaged impedance drops predicted atrial transmural lesions, their predictive performance was comparable to that of conventional measures. This study further demonstrated that averaged impedance drops significantly improved lesion size estimation compared with unfiltered impedance drops, although the correlation was modest. Given that the combination of impedance drop and RF energy showed a stronger correlation with lesion size, as we reported previously,^11^ further studies are warranted to determine whether averaged impedance drops provide superior lesion size prediction when combined with RF energy.

**Figure 6:**
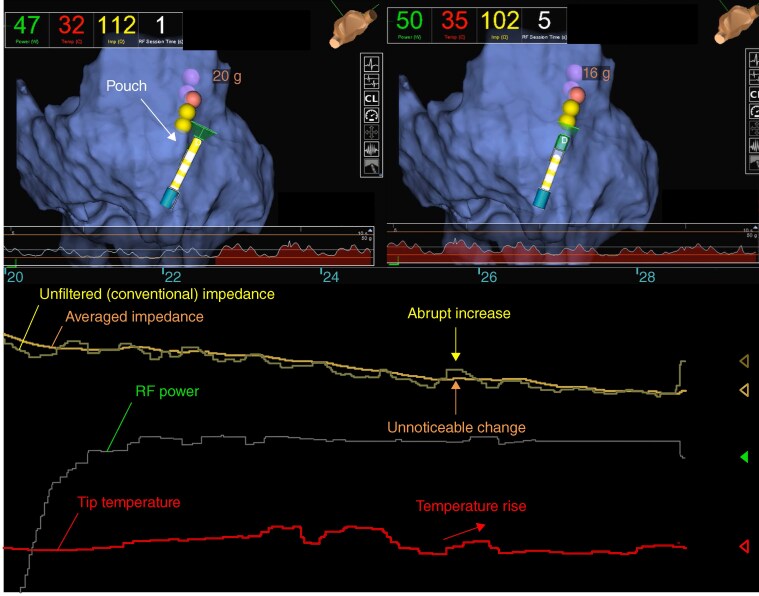
Representative case showing dissociation between unfiltered and averaged impedance curves during RF ablation. The top panels show three-dimensional electroanatomical maps with the ablation catheter positioned outside (*left*) and inside (*right*) the pouch. The lower panel presents the unfiltered impedance (yellow line), averaged impedance (orange line), RF power (green line), and tip temperature (red line). When the ablation catheter was incidentally migrated into the pouch (*top right*), the unfiltered impedance demonstrated an abrupt increase despite minimal change in the averaged impedance values, followed by a rise in tip temperature (*lower panel*).

Conventional impedance changes during RF application includes cardiac and respiratory oscillation artifacts in addition to the actual physiological reaction of myocardial tissue to RF energy, potentially leading to over- or under-estimations of RF lesion formation and the risk of SPs. In contrast, averaged impedance measurements theoretically suppress physiological artifacts by filtering out oscillations in the raw impedance signal. As a result, averaged impedance drops may more accurately represent the true myocardial response to RF energy delivery, potentially accounting for their superior predictive performance compared with unfiltered impedance drops. Nevertheless, a sudden change in the unfiltered impedance values without a corresponding shift in the averaged impedance trace during RF application may indicate subtle catheter migration into anatomical recesses such as pouches or diverticula (*Figure 6*). This observation underscores the complementary value of concurrently monitoring both unfiltered and averaged impedance curves.

### Rate of impedance decline and other parameters for predicting SPs

The evidence regarding the association between the rate of impedance drop and SP occurrence is limited. Nguyen et al. reported that rapid initial impedance reduction during RF application was significantly associated with SP occurrence using the ThermoCool SmartTouch™ SF ablation catheter in both ex vivo and porcine thigh muscle models.^25^ However, among lesions with greater impedance drops, the duration required to reach the initial 10%, 15%, and 20% reductions in the averaged %Imp-drop was not associated with SPs. Several factors may explain this discrepancy. First, we used the TactiFlex™ SE ablation catheter, which has been generally reported to have a lower incidence of SPs compared with other open-irrigated ablation catheter, possibly due to the catheter design, including a 4.0-mm tip, temperature monitoring via a thermocouple located at 0.3 mm from the distal tip, and efficient irrigation through laser-cut kerfs used under the temperature guided power-regulation.^7^ Of 959 lesions, 20–25% with and without SPs reached the maximum temperature of 43 °C (*Table 1*), indicating that temperature-controlled power titration was engaged during those applications. This feedback may mitigate the early rise in tissue temperature at the onset of RF applications, while it does not occur when using a power-controlled ablation catheter (ThermoCool SmartTouch™ SF). Second, this study employed an *in vivo* swine beating-heart model, which differs markedly from the thigh muscle model in several key aspects that influence SP formation. In the *in vivo* setting, dynamic blood-flow, catheter stability, and native myocardial architecture interact to modulate tissue heating during RF energy delivery, thereby affecting the incidence of SPs,^26–29^ but providing a more physiologically relevant simulation of clinical conditions, compared with the ex-vivo or thigh-muscle model. Our findings suggest that an initial rapid decline in impedance during RF application does not necessarily indicate an increased risk of SPs when a temperature-controlled ablation catheter is used.

Notably, all SPs that occurred during RF applications at power settings at 40 W or less and within 90 s were observed either during LV ablation under reduced systolic blood pressure or during RV ablation. To the best of our knowledge, no prior studies have identified low systemic blood pressure as a potential risk factor for SPs. Given that hypotension may reflect reduced cardiac output and blood-flow stagnation adjacent to the endocardial surface, the consequent impairment in convective heat dissipation could plausibly contribute to excessive tissue heating and steam formation. While such extreme conditions are uncommon in routine clinical procedures, they may be encountered in hemodynamically unstable patients, such as those with incessant ventricular tachyarrhythmias or severe heart failure, and during veno-arterial extracorporeal membranous oxygenation support, which increases cardiac afterload. Further research is warranted to elucidate the relationship between systemic hemodynamics and SP risk. Additionally, Seiler et al. previously reported a higher incidence of SPs in the RV than in the LV in human subjects using open-irrigated catheters, which can explain the occurrence of SPs during RV applications even at relatively low RF power under stable hemodynamics.^9^

### Limitations

This study has several limitations. First, because our *in vivo* experiments were conducted in swine hearts, the findings may not translate directly to human pathology, although they were consistent with those of an ex vivo study.^6^ Second, because SPs were defined as audible pops in this study, silent SPs, which can be detected with intracardiac echocardiography, may have been missed.^26^ Finally, the proposed cut-offs and their predictive accuracies may vary depending on the catheter platforms and energy-delivery settings.

## Conclusion

Averaged %Imp-drop was strongly associated with SP occurrence and provided a more accurate assessment of lesion characteristics than unfiltered values. Averaging real-time impedance may improve procedural safety and efficacy in RF ablation.

## Supplementary Material

euaf315_Supplementary_Data

## Data Availability

The data underlying this article will be shared on reasonable request to the corresponding author.
