# Supplementary material for: Averaging real-time impedance enhances the prediction of steam pop risk and lesion characteristics
Source: Europace. 2025 Dec 9;27(12):euaf315. doi: 10.1093/europace/euaf315 (PMC12715312; doi:10.1093/europace/euaf315)
Supplement: euaf315_Supplementary_Data [file euaf315_supplementary_data.docx]

**Table S1:**

|  | **Ventricle (N=487)** | **Steam pop (-)**  N=451 | **Steam pop (+)**  N=36 | **P-values** |
| --- | --- | --- | --- | --- |
|  | Predefined RF power, n (%) | 30 W: 90 (20.0%)  35 W: 93 (20.6%)  40 W: 98 (21.7%)  45 W: 84 (18.6%)  50 W: 86 (19.1%) | 30 W: 1 (2.8%)  35 W: 3 (8.3%)  40 W: 10 (27.8%)  45 W: 11 (30.6%)  50 W: 11 (30.6%) | 0.013* |
|  | Averaged RF power, W | 39.0 [33.0-44.0] | 44.0 [39.5-49.0] | <0.001* |
|  | Averaged contact force, g | 12.0 [10.0-16.0] | 12.5 [10.0-16.0] | 0.77 |
|  | Initial Imp, Ω | 108 [102-114] | 105 [103-110] | 0.08 |
|  | Averaged initial Imp, Ω | 106 [101-113] | 104 [100-110] | 0.18 |
|  | Imp-drop, Ω | 21.0 [18.0-26.0] | 27.5 [24.0-30.5] | <0.001* |
|  | Averaged Imp-drop, Ω | 18.3 [13.8-22.7] | 25.1 [22.6-28.1] | <0.001* |
|  | %Imp-drop, % | 20.0 [17.0-24.0] | 26.5 [24.0-28.0] | <0.001* |
|  | Averaged %Imp-drop, % | 17.0 [13.2-21.2] | 24.1 [22.2-26.6] | <0.001* |
|  | Duration, sec | 43.0 [24.0-82.0] | 62.5 [38.5-88.5] | 0.039* |
|  | Energy, J | 1772 [905-2987] | 2581 [1726-3658] | 0.002* |
|  | Averaged temperature, °C | 35.0 [33.0-38.0] | 36.0 [34.0-38.0] | 0.33 |
|  | Reach maximum temperature, n (%) | 95 (21.1%) | 8 (22.2%) | 0.90 |

Abbreviations: RF, radiofrequency; Imp, impedance. **P* < 0.05.

**
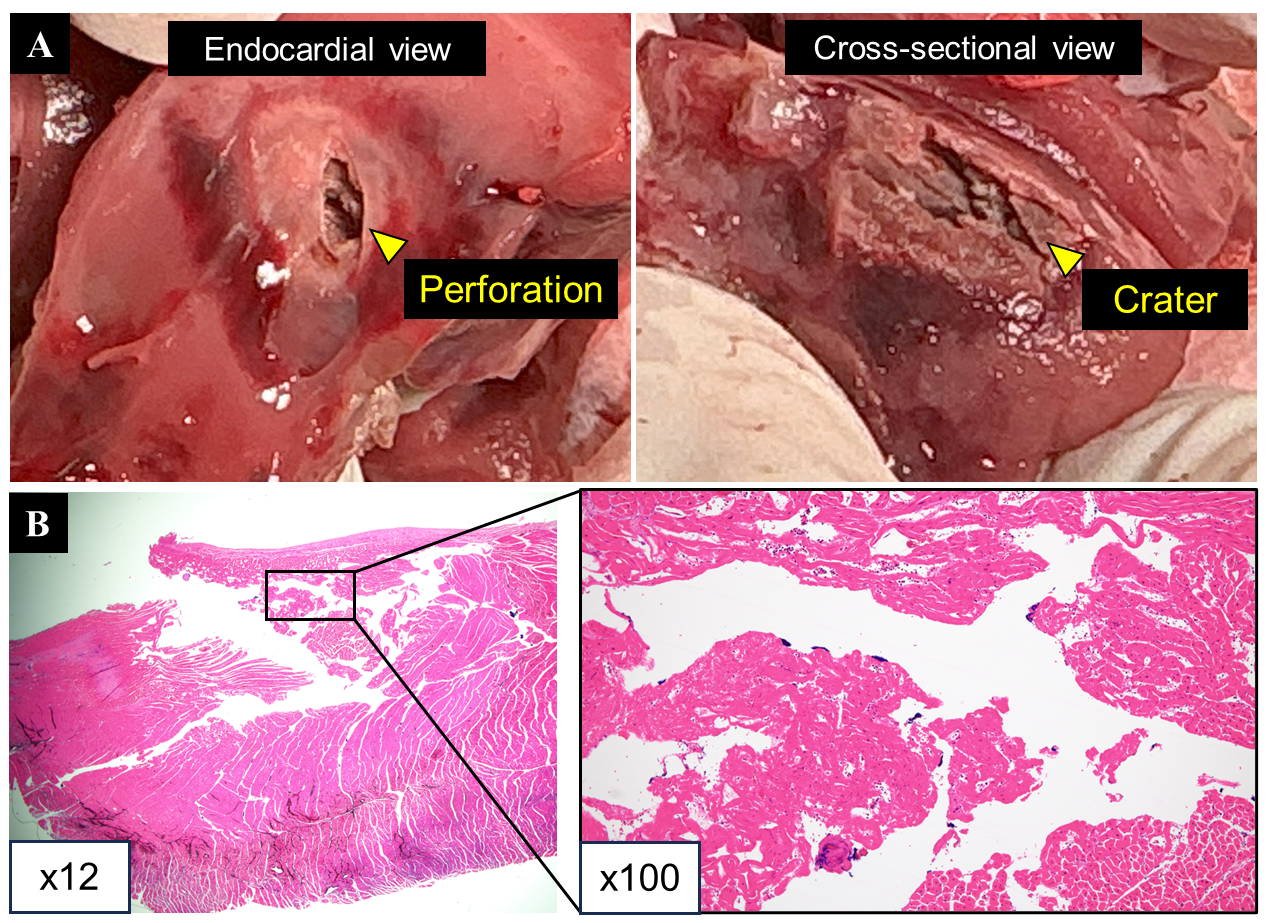
Figure S1:** Representative lesion with steam pop. **(A)** Macroscopic assessment shows tissue perforation in the endocardial view (*left*) and crater formation in the cross-sectional view (*right*). **(B)** Histopathological analysis demonstrates tissue destruction, extensive coagulation necrosis, and cellular dissolution.


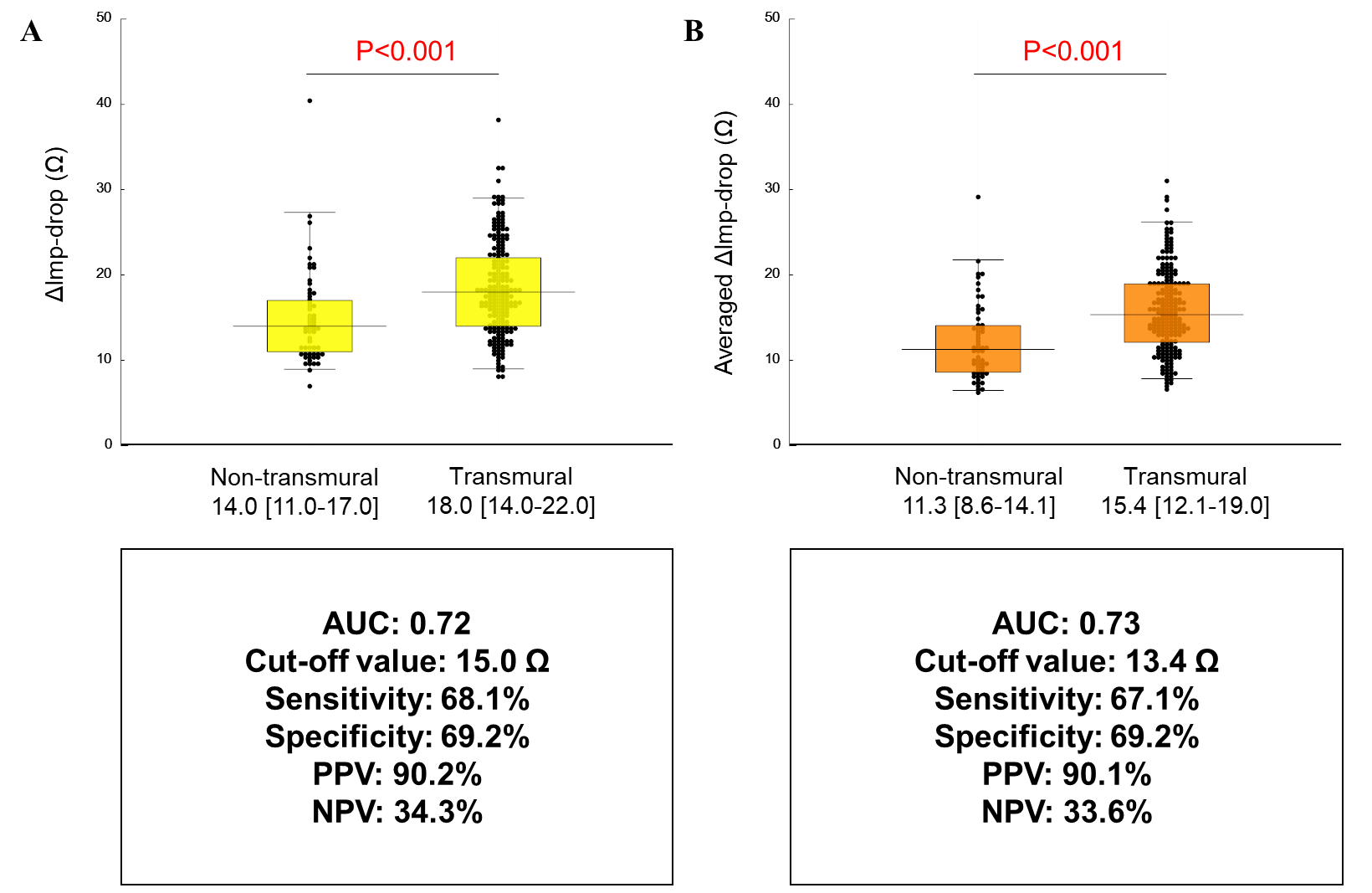
**Figure S2:** Unfiltered and averaged impedance drops in non-transmural and transmural lesions. Box plots illustrate the distribution for **(A)** unfiltered ΔImp-drop and **(B)** averaged ΔImp-drop. The area under the receiver operating characteristic curve (AUC), optimal cut-off values, sensitivity, specificity, positive predictive value (PPV), and negative predictive value (NPV) are shown below each plot.


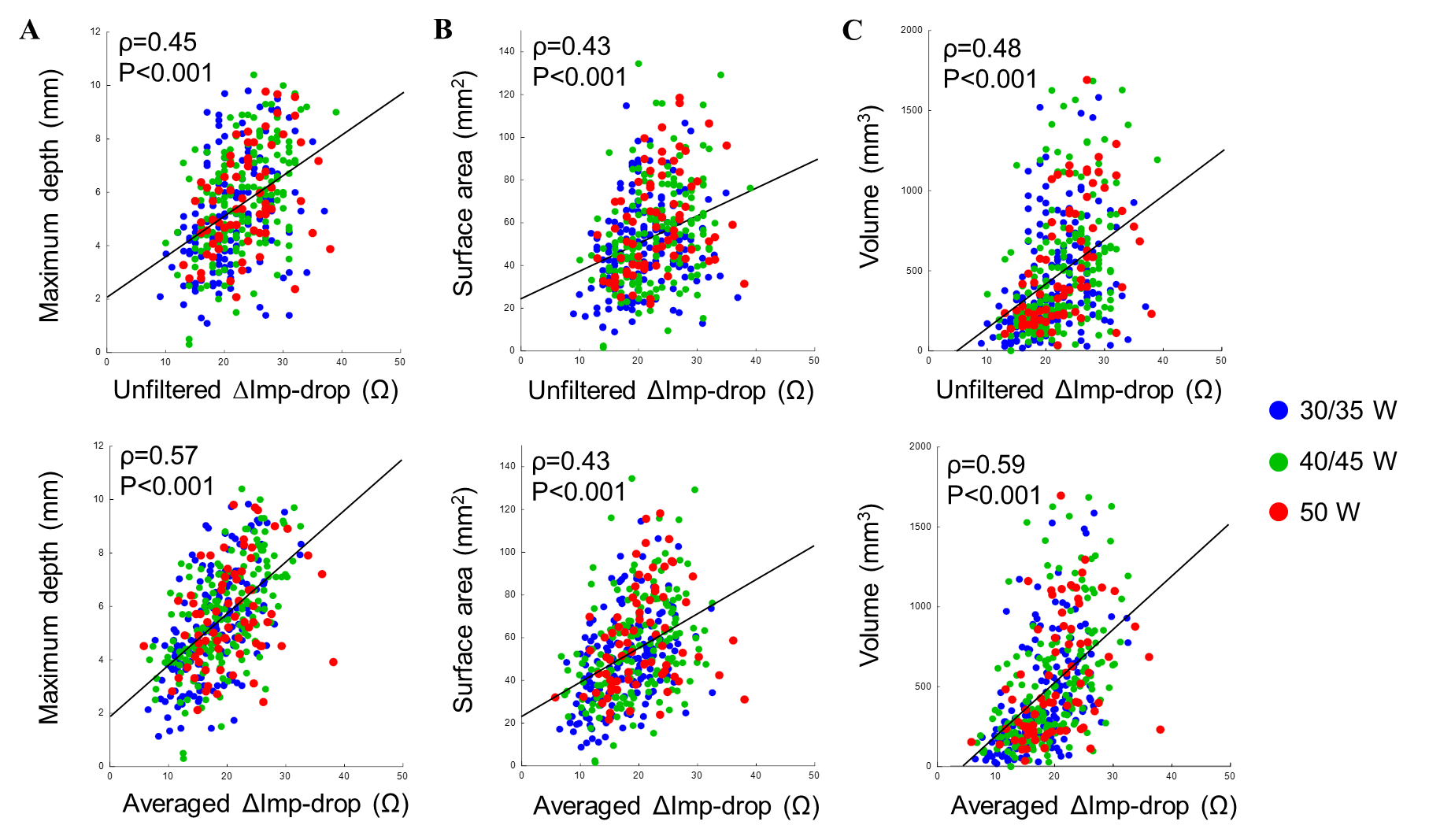
**Figure S3:** Correlations of unfiltered (*upper*) and averaged (*lower*) ΔImp-drop with lesion metrics. Scatter plots show relationships with maximum depth **(A)**, surface area **(B)**, and volume **(C)**. Blue, green, and red dots represent lesions created with radiofrequency power of 30-35 W, 40-45 W, and 50 W, respectively.
